# Integration of Transcriptomics and Metabolomics Reveals Mechanisms of High-Temperature Stress Tolerance in the Hepatopancreas of *Penaeus monodon*

**DOI:** 10.3390/biology14060591

**Published:** 2025-05-23

**Authors:** Li Liu, Peng Zhao, Lishi Yang, Yundong Li, Zhong Huang, Qibin Yang, Yukai Yang, Jianzhi Shi, Yibiao Chen, Jianhua Huang

**Affiliations:** 1 South China Sea Fisheries Research Institute, Chinese Academy of Fishery Sciences/Key Laboratory of South China Sea Fishery Resources Exploitation and Utilization, Ministry of Agriculture and Rural Affairs, Guangzhou 510300, China; liuli5850315@163.com (L.L.); 15383644216@163.com (P.Z.); yangls2016@163.com (L.Y.); liyd2019@163.com (Y.L.); shijianzhi1989@163.com (J.S.); 2 College of Fisheries and Life Science, Shanghai Ocean University, Shanghai 201306, China; 3 Tropical Fishery Research and Development Center, South China Sea Fisheries Research Institute, Chinese Academy of Fishery Sciences, Sanya 572018, China; yangqibin1208@163.com; 4 Shenzhen Base of South China Sea Fisheries Research Institute, Chinese Academy of Fishery Sciences, Shenzhen 518108, China; huangzhongnhs@163.com (Z.H.); yangyukai1986@163.com (Y.Y.); ly-164@163.com (Y.C.)

**Keywords:** *Penaeus monodon*, high-temperature stress, energy supply, immune regulation, amino acid metabolism, glutathione metabolism

## Abstract

High temperatures in summer often trigger disease outbreaks in shrimp, resulting in significant economic losses. To investigate the heat tolerance mechanisms of *Penaeus monodon*, juvenile tiger shrimp were subjected to a high-temperature stress of 38 °C for 144 h. The cumulative survival rate of shrimp sharply decreased to 5.29% in the later 144 h. The heat-sensitive shrimps (S group) were collected in the first 24 h, while those that survived beyond 120 h were collected as the heat-tolerant group (T group). The hepatopancreas of two groups was subjected to transcriptomic and metabolomic analysis. The results revealed that, compared to the S group, the T group exhibited a total of 3527 DEGs, including 2199 upregulated and 1328 downregulated genes. Additionally, 353 DAMs were identified in the T group, with 75 metabolites showing increased levels and 278 metabolites displaying decreased levels. The results revealed that the mechanisms of heat tolerance involve energy supply strategies, immune system regulation, amino acid metabolism, and glutathione metabolism. Energy supply strategies include the digestion and absorption of carbohydrates and proteins, glycolysis/gluconeogenesis, fructose and mannose metabolism, and pyruvate metabolism, all of which collectively meet energy demands in high-temperature environments. The immune system is regulated by C-type lectin receptor pathways and IL-17 signaling pathways, which together coordinate innate immunity to prevent pathogen invasion. In amino acid metabolism, various glycogenic amino acids, such as histidine, phenylalanine, valine, and serine, are metabolized for energy, while excess ammonia is converted to γ-glutamyl-glutamate and L-glutamate to mitigate ammonia accumulation. Combined transcriptomic and metabolomic analyses further indicate that glutathione metabolism plays a crucial role in the adaptation of *P. monodon* to high-temperature environments. This study explains the high-temperature tolerance mechanism of *P. monodon* from the aspects of gene expression regulation and material metabolism regulation and also provides a scientific basis and basic data for the selection and breeding of new varieties of *P. monodon* with a high-temperature tolerance.

## Simple Summary

This study identified heat-sensitive and heat-tolerant individuals of *Penaeus monodon* juveniles under acute high-temperature stress conditions at 38 °C and elucidated the thermal adaptation mechanisms through a comparative analysis of differential gene expression and molecular metabolism between these two phenotypic groups. The results demonstrate that the thermoadaptive mechanism in *P. monodon* involves a complex regulatory network, where multiple biological processes, including energy supply strategies, immune system modulation, amino acid metabolism, and glutathione metabolism, coordinately contribute to thermal resistance. These findings provide a theoretical foundation for breeding thermally resistant strains and the genetic improvement of *P. monodon* through selective cultivation.

## 1. Introduction

Global climate warming is leading to rising sea temperatures, significantly threatening the survival and growth of marine animals, even resulting in the extinction of certain species, disrupting regional biodiversity and community structures, and affecting the distribution or structure of marine animal populations [1,2].

Acute or long-term changes in environmental temperature will damage the physiological status of fish and inhibit their growth. As the environment changes, organisms also adapt passively. Studies have shown that different species and individuals respond variably to high-temperature conditions. High-temperature-sensitive and tolerant individuals were naturally occurring in cold-water fish like rainbow trout [3]. Temperature acclimation (TA) can increase mitochondrial plasticity and improve the high-temperature tolerance of rainbow trout [4]. Additionally, the ability to tolerate high temperatures was found to be inherited by offspring in zebrafish [5]. Organisms adapt to high temperatures by altering the expression of relevant genes and metabolic levels, which influences aquatic animal behavior, physiology (such as heart rate), and biochemistry (including protein and lipid structure and expression) [6,7]. According to these studies, it is necessary to conduct genetic improvements in the later stages to enhance species’ heat adaptation based on research on heat adaptation.

The high temperature of water impacts tissue structure, enzyme activity levels, transcriptional regulation, and metabolism. Cyclic high-temperature stress-induced damage to the gill tissues of *Litopenaeus vannamei* triggers an oxidative stress response. Various biomarkers of serum, including total antioxidant capacity (T-AOC), total superoxide dismutase (T-SOD), glutamate dehydrogenase (GDH), glutaminase (GLS), hexokinase (HK), and acid phosphatase (ACP), were significantly changed [8,9]. Furthermore, endoplasmic reticulum stress, cell apoptosis, and impaired osmoregulatory function were also observed [10]. Research indicates that the metabolic demands of microorganisms such as bacteria and algae increase substantially in high-temperature environments, leading to the excessive consumption of oxygen in the water [11]. Moreover, the accumulation of reactive oxygen species (ROS) and elevated levels of heat shock proteins (HSPs) trigger transcription factors such as heat shock transcription factor 1 (HSF1), hypoxia-inducible factor 1α (HIF-1α), and tumor suppressor protein (p53), which affect energy supply, immune response, and metabolic processes in marine animals [12,13]. These processes deplete the innate and adaptive immune and antioxidant systems of fish and shrimp, increasing their susceptibility to pathogens [14].

With the rapid development of biological big data analysis methods, the joint analysis of multi-omics technologies, for example, transcriptomics and metabolomics, is increasingly applied in the study of the mechanisms of organisms. Transcriptomic analyses show that thermal stress suppresses protein synthesis in juvenile grass carp, reduces fatty acid synthesis, and weakens carbohydrate metabolism [15]. Additionally, transcriptomic analyses reveal significant differences in energy allocation among the hepatopancreas, gills, and muscle of *L. vannamei* under thermal stress [16]. miR-301b-5p and its target gene nfatc2ip were reported to regulate the inflammatory response in the liver of rainbow trout (*Oncorhynchus mykiss*) under high-temperature stress, facilitating the repair of microstructural liver damage induced by thermal stress [3]. Transcriptomic analyses confirmed that immune pathways and metabolic pathways are essential mechanisms for *Procambarus clarkii* to cope with high and low-temperature stress [17].

Metabolomics provides insights into the response mechanisms of organisms to thermal stress by analyzing low molecular weight metabolites in cells, tissues, or biological fluids [18]. Levels of adenosine, inosine, xanthine, guanosine, and deoxyguanosine, which are critical for DNA/RNA synthesis, are markedly reduced, while the abundance of D-glucose 6-P significantly increases [19]. High-temperature stress significantly disrupts the metabolism of glycerophospholipids and fatty acids, leading to membrane damage and lipid metabolic disorders in the liver cells of rainbow trout [20]. Integrated metabolomic and transcriptomic analyses revealed that enhanced amino acid and carbohydrate metabolism, along with inhibited fatty acid metabolism, constitute a potential energy conservation strategy for sturgeon *Acipenser dabryanus* during high-temperature stress [21]. The same integrated analysis demonstrated that gene expression and metabolite levels of lipid homeostasis were varied, thereby alleviating lipid metabolic disorders induced by thermal stress in turbot (*Scophthalmus maximus*) [22]. All of this research showed that multi-omics technologies has been powerful in analyzing molecular mechanisms.

*Penaeus monodon*, commonly known as the black tiger shrimp, is one of the major cultivated shrimp species worldwide, with advantages in rapid growth, high-quality meat, strong disease resistance, and low production costs. It is widely farmed in tropical and subtropical regions such as China, Southeast Asia, and Africa, and has a broad temperature tolerance, ranging from 15 to 35 °C, with an optimal temperature of 25 to 30 °C [23]. However, extremely high temperatures in summer still challenge the farming of *P. monodon*, which suffers greater losses during disease outbreaks, such as White Spot Syndrome (WSS), Early Mortality Syndrome (EMS), Acute Hepatopancreatic Necrosis Disease (AHPND), Enterocytozoon Hepatopenaei (EHP), and so on [24,25,26,27]. Moreover, extremely high temperatures deteriorate the water quality, causing eutrophication and imbalances in ammonia nitrogen, often leading to disruptions in the microbial communities of the farming ponds, which significantly impact physiological and biochemical parameters and growth performance, and increase mortality [28]. Uncovering the molecular mechanisms upon high-temperature stress, and further promoting genetic improvements to cope with extreme summer environments, have become urgent tasks in black tiger shrimp aquaculture.

Based on the preliminary experimental results, the number of shrimp surviving under 38 °C stress was sufficient to ensure an adequate sample size for subsequent tests. In this paper, we characterized temperature-sensitive and heat-tolerant individuals of the *P. monodon* under 38 °C high-temperature stress for 144 h. We compared the differences in gene expression and metabolism levels and then identified key genes and metabolic markers associated with high-temperature tolerance by integrated transcriptomic and metabolomic analyses. The results provide useful data for the strategies for dealing with high-temperature threats in the shrimp industry, as well as benefits the selective breeding of high-temperature-tolerant species of tiger shrimp.

## 2. Materials and Methods

### 2.1. Shrimp and Rearing Conditions

Healthy juvenile *P. monodon* (average weight 4.97 ± 1.95 g) were sourced from the Shenzhen Experimental Base of the South China Sea Fisheries Research Institute in Shenzhen city, Guangdong Province in China. They were acclimated in a concrete pond for 7 days under continuous aeration, with a maintained temperature of 30 ± 2 °C and salinity at 30 ppt, and fed commercial feed three times daily. This study was approved by the Animal Care and Use Committee of the South China Sea Fisheries Research Institute, Chinese Academy of Fishery Sciences (Approval No. nhdf2025-10). All sampling procedures and experimental protocols were conducted in strict accordance with the committee’s established guidelines and regulations.

### 2.2. High-Temperature Stress and Sample Collection

Following the acclimatization period, a total of 700 juvenile *P. monodon* were introduced into experimental tanks (length 5.5 m, width 2.85 m, water depth 1 m). Water temperature was gradient heated with a speed of 2 °C per day, starting from an initial temperature of 30 °C, until the target temperature of 38 °C was reached. During the experiment, the tanks were monitored every 20 min to remove moribund shrimp (exhibiting signs of lateral recumbency and minimal response to touch), which were recorded. The experiment lasted for 144 h. The shrimp that were moribund during the first 24 h were considered the heat-sensitive group (S group), and those that survived beyond 120 h were classified as the heat-tolerant group (T group).

Currently, there is no established anesthesia method for *Penaeus monodon*. To avoid compromising the experimental results, live sampling was employed in this study. The hepatopancreas of each shrimp was dissected into two parts. One half was placed in RNAlater solution (Ambion, Austin, TX, USA) for transcriptomic analysis. The other half was rapidly frozen in liquid nitrogen for metabolomic analysis. In the transcriptomic analysis, twelve shrimp were included per group, with hepatopancreas tissues from every four individuals pooled to form one test sample. For the metabolomic analysis, 20 shrimp were included per group, and similarly, hepatopancreas tissues from every 4 individuals were combined to constitute a single test sample.

### 2.3. -Transcriptomic Analysis

#### 2.3.1. RNA Extraction and Library Construction

Total RNA was extracted from the hepatopancreas using TRIzol reagent according to the manufacturer’s instructions. RNA purity and quantification were assessed using a NanoDrop 2000 spectrophotometer (ThermoScientific, Wilmington, DE, USA), while RNA integrity was evaluated using an Agilent 2100 Bioanalyzer (Agilent Technologies, Santa Clara, CA, USA). The transcriptomic library was constructed using the VAHTS Universal V5 RNA-seq Library Prep Kit following the manufacturer’s protocol. Sequencing of the library was performed on the Illumina Novaseq 6000 platform, generating 150 bp paired-end reads.

#### 2.3.2. Bioinformatics Analysis of the Transcriptome

Raw reads in fastq format were processed using fastp 0.20.1 software to remove low-quality reads, resulting in clean reads. Subsequently, clean reads were aligned and annotated against the *P. monodon* genome using HISAT2 2.1.0 software, and gene expression levels were calculated as FPKM (fragments per kilobase of transcript per million mapped reads). Read counts for each gene were obtained using HTSeq-count. PCA analyses were performed using R (v 3.2.0) to evaluate the biological duplication of samples. Differential expression analysis was performed with DESeq2 software, identifying differentially expressed genes (DEGs) based on the criteria of q-value < 0.05 and fold change > 2 or fold change < 0.5. A hierarchical cluster analysis of DEGs was performed using R (v 3.2.0) to demonstrate the expression pattern of genes in different groups and samples. GO and KEGG pathway enrichment analyses for DEGs were conducted using hypergeometric tests based on the Gene Ontology (GO) database and the Kyoto Encyclopedia of Genes and Genomes (KEGG) database. The transcriptional characteristics of immune- and oxidative stress-related DEGs were further analyzed.

#### 2.3.3. qRT-PCR Verification

Total RNA extracted from the hepatopancreas of each group was used as a template for cDNA synthesis by the Evo M-MLV Plus cDNA kit (AG, Changsha, China). We randomly selected 12 differentially expressed genes for validation through qRT-PCR to verify the transcriptomic sequencing results, using the EF-1α gene (GenBank MG775229.1) as an internal reference. The qPCR was conducted using the LightCycler^®^ 480 II (Roche, Mannheim, Germany), following the method by R. Fan et al. [29]. Each sample and reference gene was analyzed in triplicate, and data were calculated using the 2^−ΔΔCt^ method.

### 2.4. Metabolomics Analysis

#### 2.4.1. Metabolite Extraction and LC-MS Analysis

A total of 30 mg of hepatopancreas was mixed with 400 μL of methanol–water (*v*:*v* = 4:1, containing mixed internal standards at 4 μg/mL). After pre-cooling at −40 °C for 2 min, the mixture was homogenized for 2 min. Ultrasonic extraction was performed in an ice-water bath for 10 min, followed by centrifugation at 12,000 rpm for 10 min at 4 °C. The supernatant (300 μL) was collected and evaporated to dryness in a sample vial for LC-MS analysis. Prior to the analysis, the residue was reconstituted with 300 μL methanol–water (*v*:*v* = 1:4), sonicated for 3 min in an ice-water bath, and then left to stand at −40 °C for 2 h. The sample was centrifuged again at 12,000 rpm for 10 min at 4 °C.

The LC-MS analysis was conducted using a Waters ACQUITY UPLC I-Class Plus coupled with a Thermo QE ultra-high-performance liquid chromatography–tandem mass spectrometry system. Liquid chromatographic separation was performed on an ACQUITY UPLC HSS T3 column (100 mm × 2.1 mm, 1.8 μm) at a column temperature of 45 °C, using mobile phase A (water with 0.1% formic acid) and B (acetonitrile) at a flow rate of 0.35 mL/min with an injection volume of 3 μL. Mass spectrometry utilized an ESI ion source with separate scanning for positive and negative ions, employing a data-dependent acquisition (DDA) mode.

#### 2.4.2. Bioinformatics Analysis of the Metabolome

The raw data were processed using the metabolomics software Progenesis QI v3.0 (Nonlinear Dynamics, Newcastle, UK) for baseline filtering, peak identification, integration, retention time correction, peak alignment, and normalization. Metabolites were identified and annotated using the Human Metabolome Database (HMDB), Lipidmaps (v2.3), the METLIN database, and the LuMet-Animal 3.0 local database. Orthogonal Partial Least Squares Discriminant Analysis (OPLS-DA) was applied to differentiate the overall metabolic profiles between groups. Differential metabolites were selected based on the combination of multivariate and univariate analyses (VIP > 1 and *p*-value < 0.05). The volcano plot was employed to visualize the p-values, VIP (Variable Importance in Projection) scores, and FC (fold change) values. Hierarchical clustering was subsequently performed to analyze the expression levels of selected significantly differential metabolites. A KEGG pathway enrichment analysis for the differential metabolites was conducted using hypergeometric testing based on the KEGG PATHWAY and KEGG ORTHOLOGY databases. The metabolite biomarkers were further identified.

### 2.5. Combined Analysis of Hepatopancreatic Transcriptome and Metabolome

After filtering the data for differentially abundant metabolites (DAMs) and differentially expressed genes (DEGs), a correlation analysis was performed using the Spearman algorithm to construct a correlation heatmap for DEGs and differentially expressed metabolites (DEMs). A joint analysis was conducted on the KEGG enrichment results from both transcriptomics and metabolomics, focusing on pathways that were significantly enriched (*p*-value < 0.05) in each dataset. Commonly enriched KEGG pathways were extracted, and the results were visualized using a bubble plot.

## 3. Results

### 3.1. The Cumulative Survival Rate of P. Monodon

**Figure 1:**
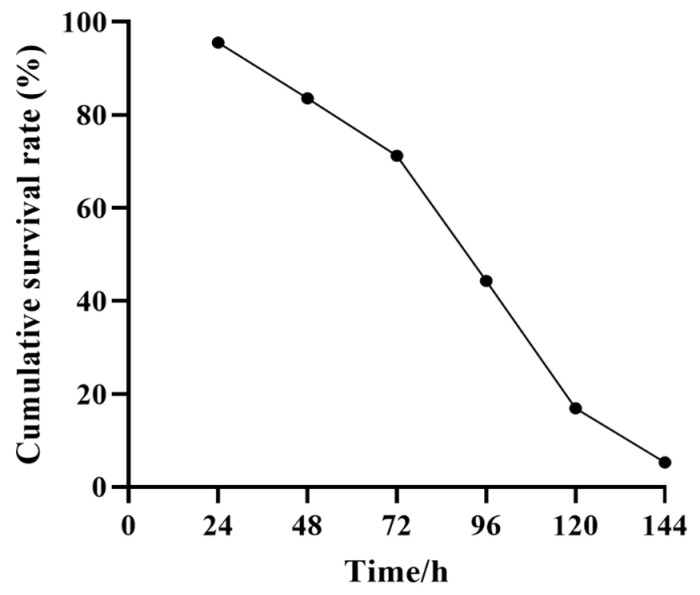
Cumulative survival rate of *P. monodon* under high-temperature stress.

Typical signs of stressed shrimp, such as reduction in food consumption and red coloration, were observed, and the dead shrimps were recorded throughout the whole experimental stage. High-temperature stress threatened the survival of shrimp, and the cumulative survival within 24 h was 95.57%, and then sharply decreased to 5.29% in the later 144 h, accompanied with a rise in the temperature. The peak mortality period occurred between 72 and 120 h (Figure 1). Consequently, shrimp that succumbed within 24 h were classified as heat-sensitive (S group), whereas those that survived beyond 120 h were classified as heat-tolerant (T group).

### 3.2. Hepatopancreas Transcriptomic Analysis

#### 3.2.1. Identification and Functional Annotation of the DEGs

**Figure 2:**
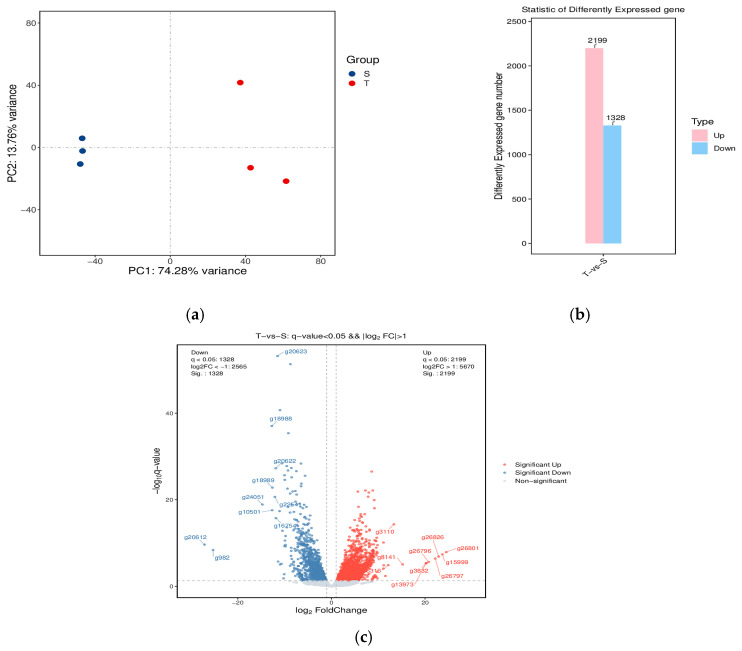
Differential genes in the hepatopancreas of *P. monodon* under high-temperature stress. (**a**) PCA diagram; (**b**) histogram of differentially expressed gene statistics; and (**c**) volcano map of transcriptome genes.

The PCA analysis in this study revealed a distinct clustering trend between the T group and S group (Figure 2a). Compared to the S group, a total of 3,527 DEGs were identified in the T group, including 2199 upregulated and 1328 downregulated genes (Figure 2b,c).

**Figure 3:**
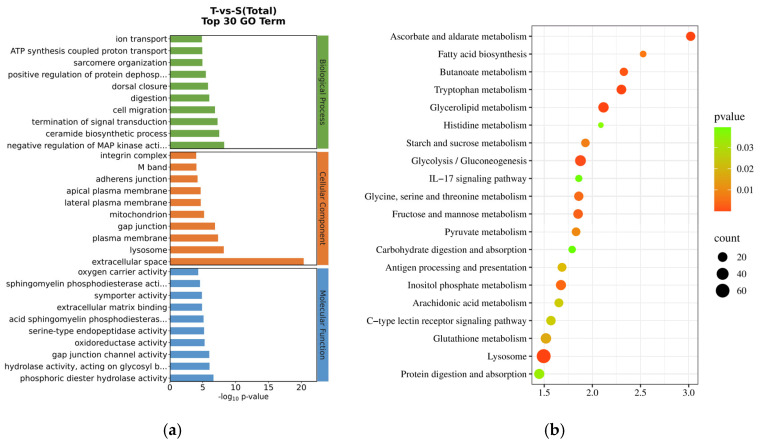
The pathway enrichment in the hepatopancreas of *P. monodon* under high-temperature stress. (**a**) GO enrichment terms; (**b**) KEGG enrichment terms.

The further analysis of the DEGs through GO and KEGG enrichment revealed that 448 GO terms were significantly enriched between the two groups, comprising 273 in biological processes, 67 in cellular components, and 108 in molecular functions. The functional enrichment of the DEGs included categories such as oxidoreductase activity, the negative regulation of MAP kinase activity, integrins, and mitochondrial functions (Figure 3a). The KEGG enrichment analysis indicated that there were 60 significantly differentially enriched KEGG pathways between the T and S groups, including pathways related to glutathione metabolism, lysosome, carbohydrate digestion and absorption, protein digestion and absorption, glycolysis/gluconeogenesis, fructose and mannose metabolism, pyruvate metabolism, glycine, serine, and threonine metabolism, the C-type lectin receptor signaling pathway, and the IL-17 signaling pathway (Figure 3b).

In the glutathione metabolism pathway, the expression of genes such as *GLCL* and *Ggct* was upregulated, while genes like *GST1*, *ICDH*, and *Rrm1* were downregulated. Within the lysosome pathway, genes including *MLA1* and *CTSC* were upregulated, whereas *NPC1*, *CTSD*, and *GLB1* showed downregulation. In the glycolysis/gluconeogenesis pathway, genes such as *CG10160* and *TEgg056i02.1* were upregulated, while *G6pt*, *ALDH3*, and *Aldhx* were downregulated. For the glycine, serine, and threonine metabolism pathway, *Tdh* and *LPIPOX* exhibited upregulation, while *bhmt*, *DAO*, and *AGT1* were downregulated. In the C-type lectin receptor signaling pathway, *COX2*, *CG12559*, and *FYN* were upregulated, while *DDB_G0277917* and *MLT* were downregulated. Lastly, in the IL-17 signaling pathway, *CG11992 (Relish)*, *mapk15*, *Traf6,* and *CG5680 (JNK)* showed upregulation, whereas the expression of the *T05E11.3 (Hsp90b1)* gene was downregulated (Table 1).

#### 3.2.2. Analysis of Specific Genes for Immunity and Oxidative Stress

In addition, some genes related to immunity and oxidative stress were further analyzed. Immune-related genes, including anti-lipopolysaccharide factor isoform 6 (*ALF*), serine proteinase inhibitor 6 (*Serpinb1a*), beta-integrin (*ZK1058.2*), and sushi, von Willebrand factor type A, EGF, and pentraxin domain-containing protein 1-like (*Svep1*), were found to be upregulated, while i-type lysozyme-like protein 1 (*lysoz*), Kazal-type serine proteinase inhibitor 1 (*Agrin*), gamma-interferon-inducible lysosomal thiol reductase (*ifi30*), and chitotriosidase-1-like (*Chia*) were downregulated.

**Figure 4:**
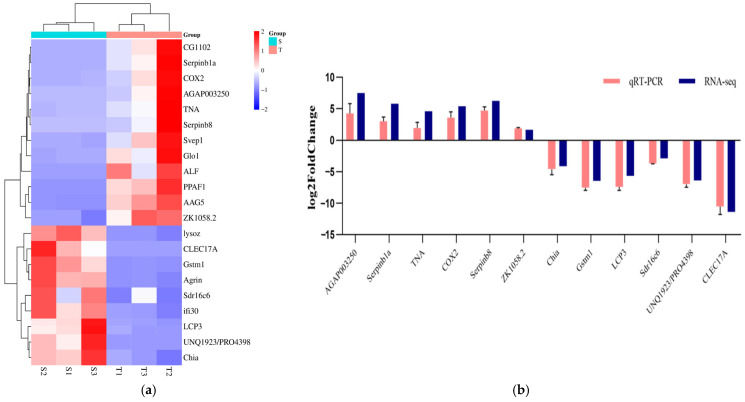
The transcription characteristics of gene markers and differential gene validation of *P. monodon* under high-temperature stress. (**a**) Heatmap of differential genes; (**b**) qRT-PCR differential gene validation.

For oxidative stress and antioxidant-related genes, lactoylglutathione lyase (*Glo1*), prophenoloxidase-activating enzyme 2a (*PPAF1*), and cyclooxygenase (*COX2*) were upregulated, whereas short-chain dehydrogenase/reductase family 16C member 6-like (*Sdr16c6*) and glutathione S-transferase (*Gstm1*) were downregulated (Figure 4a). The qRT-PCR validation of the transcriptomic sequencing results indicated that the expression patterns of the genes tested by qRT-PCR were consistent with those obtained from RNA-Seq, confirming the reliability and accuracy of the RNASeq analysis (Figure 4b).

### 3.3. Hepatopancreas Metabolomics Analysis

#### 3.3.1. Identification and Functional Analysis of Differential Metabolites

**Figure 5:**
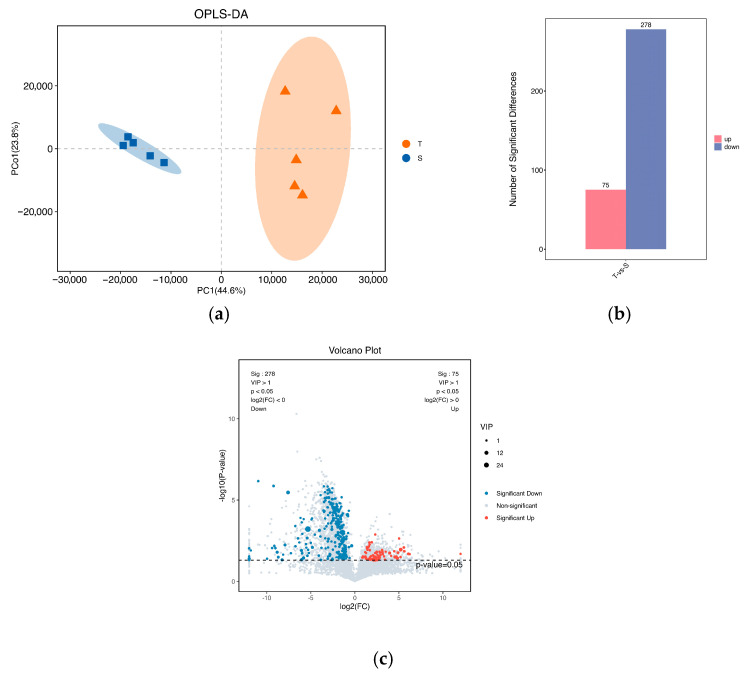
Metabolic pattern variation in the hepatopancreas of *P. monodon* under high-temperature stress. (**a**) OPLS–DA scatter diagram; (**b**) histogram of differential metabolites; and (**c**) volcanic map of differential metabolites.

The OPLS-DA analysis demonstrated significant differences between the T group and S group, indicating substantial alterations in the metabolites of heat-tolerant *P. monodon* under high-temperature stress (Figure 5a). Compared to the S group, a total of 353 differentially abundant metabolites were identified in the T group, with 75 metabolites showing increased expression and 278 metabolites showing decreased expression (Figure 5b,c).

**Figure 6:**
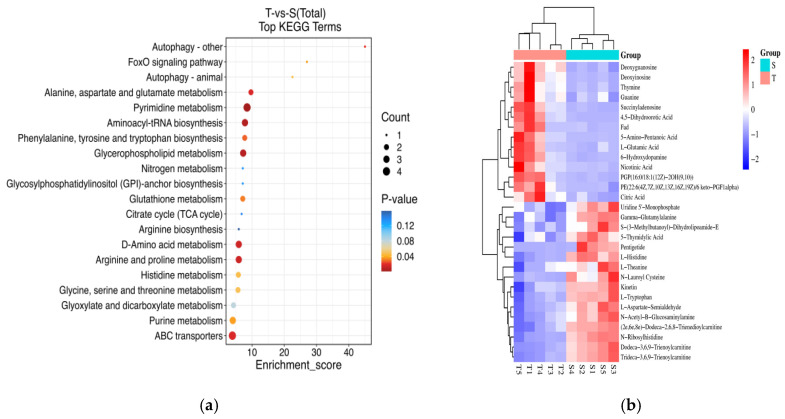
The pathway enrichment and the identification of metabolite markers in the hepatopancreas of *P. monodon* under high-temperature stress. (**a**) KEGG enrichment terms; (**b**) heatmap of differential metabolites.

The further enrichment analysis of these DAMs revealed that the primary pathways enriched between the T and S groups included amino acid metabolism (arginine, proline, glycine, serine, threonine, alanine, aspartate, and glutamate), pyrimidine and purine metabolism, glycerophospholipid metabolism, ABC transporters, autophagy, glutathione metabolism, and the FoxO signaling pathway (Figure 6a).

#### 3.3.2. Recognition of Metabolite Markers

Additionally, some stress-related marker metabolites were further analyzed, including downregulated metabolites associated with amino acid metabolism, such as L-theanine, N-ribosylhistidine, and L-tryptophan, and upregulated metabolites such as L-glutamic acid and 5-amino-pentanoic acid. In glycerophospholipid metabolism, PE (22:6 (4Z, 7Z, 10Z, 13Z, 16Z, 19Z)/6 keto-PGF1alpha) and PGP (16:0/18:1 (12Z)-2OH (9, 10)) were found to be upregulated. Nucleotide metabolism revealed the upregulation of Fad, guanine, deoxyinosine, thymine, and deoxyguanosine, while uridine 5′-monophosphate and 5-thymidylic acid were downregulated. For immune-related metabolites, pentigetide, trideca-3,6,9-trienoylcarnitine, and dodeca-3,6,9-trienoylcarnitine were downregulated, whereas 4,5-dihydroorotic acid, nicotinic acid, and 4,5-dihydroorotic acid were upregulated (Figure 6b).

### 3.4. Combined Transcriptome and Metabolome Analysis

**Figure 7:**
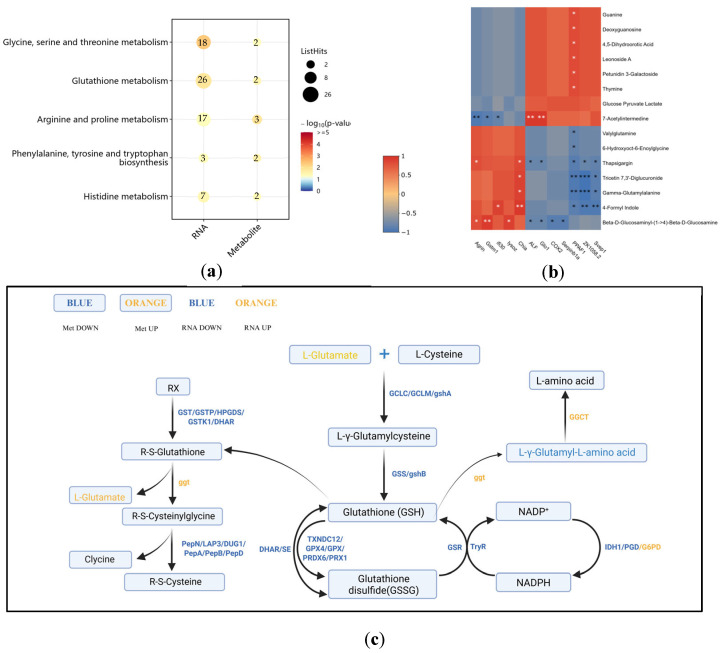
Transcriptome and metabolome correlation analysis. (**a**) KEGG pathway bubble diagram; (**b**) heatmap of differential gene–metabolite correlation, * indicates *p* < 0.05, ** indicates *p* < 0.01, and *** indicates *p* < 0.001; (**c**) The DAMs and related DEGs involved in glutathione metabolism.

The further joint analysis revealed that the pathways commonly enriched in both transcriptomics and metabolomics included glutathione metabolism, glycine, serine, and threonine metabolism, arginine and proline metabolism, biosynthesis of phenylalanine, tyrosine, and tryptophan, as well as histidine metabolism (Figure 7a). In the T group, genes such as gamma-glutamyl cyclo transferase-like (*GGCT*), glucose-6-phosphate 1-dehydrogenase (*G6PD*), and gamma-glutamyl transpeptidase (*GGT*) in the glutathione metabolism pathway were upregulated, while glutathione reductase (*GSR*), glutathione dehydrogenase/transferase (*DHAR*), leucyl aminopeptidase (*PepA*), and trypanothione-disulfide reductase (*TryR*) were downregulated. Additionally, levels of L-glutamic acid increased, while L-gamma-glutamyl-L-amino acid levels decreased (Figure 7c). The correlation analysis between metabolites and genes demonstrated strong associations between *Chia*, *PPAF1*, *ZK1058.2*, and *Svep1*, and metabolites such as Thapsigargin, Tricetin 7, 3′-Diglucuronide, Gamma-Glutamylalanine, and 4-Formyl Indole. *PPAF1* was also strongly correlated with guanine, deoxyguanosine, 4,5-dihydroorotic acid, leonoside A, petunidin 3-galactoside, and thymine. Furthermore, *Agrin*, *Gstm1*, *Lysoz*, *ALF*, *Glo1*, *COX2*, and *Serpinb1a* exhibited strong correlations with Beta-D-Glucosaminyl-(1→4)-Beta-D-Glucosamine (Figure 7b).

## 4. Discussion

Temperature is a key environmental factor for organisms that need the physiological and biochemical processes to be carried out at a certain temperature. Although shrimp belong to ectothermic animals, studies have shown that the immune and physiological activities of shrimp are significantly affected when the water temperature suddenly changes within 6~9 °C, which can even kill them. Uncovering the effector for the change in temperature and the adaptability of extreme temperature of shrimp will provide useful data for the healthy aquaculture of shrimp, as well as the breeding of new strains that resist high temperatures [23,30]. This study confirms that extreme high temperatures have lethal effects on *P. monodon*. This was accomplished by dividing the sensitive and tolerant groups and then obtaining the regulatory mechanisms of shrimp adapting to high temperatures by a joint analysis of the transcriptional regulation and metabolic levels.

In this study, the transcriptomic analysis revealed significant enrichment in glycolysis/glycogen synthesis, fructose and mannose metabolism, the IL-17 signaling pathway, the C-type lectin receptor signaling pathway, amino acid metabolism, and glutathione metabolism under high-temperature stress conditions. Therefore, it is hypothesized that the regulation of resistance and defense genes, along with the expression of oxidative and antioxidative genes, represents a comprehensive strategy for high-temperature stress resistance in *P. monodon*. The further untargeted metabolomics analysis indicated that the differentially expressed metabolites in the heat-resistant group included amino acids, peptides and their analogs, carbohydrates and their conjugates, fatty acids and conjugates, and amino acid-derived groups and their derivatives (such as aminoquinolines and related compounds). Based on the integrative analysis of transcriptomics and metabolomics, it is suggested that *P. monodon* primarily adapts to high-temperature stress by regulating immune responses, energy metabolism, various amino acid metabolic pathways, and glutathione metabolism. This study will discuss these four aspects in detail.

### 4.1. Energy Metabolism Regulation Is Necessary for the Black Tiger Shrimp to Adapt to High-Temperature Stress

Rising temperatures increase the energy demand of cellular tissues, particularly in ectothermic animals, where elevated environmental temperatures stimulate metabolic levels to rise exponentially, leading to a sharp increase in energy requirements. Increasing evidence suggests that the selective storage of energy substrates and the adjustment of energy supply mechanisms are critical strategies for organisms to cope with acute high-temperature stress [31,32,33]. Our research identified several energy supply-related pathways significantly enriched in heat-tolerant shrimp, including carbohydrate digestion and absorption, protein digestion and absorption, glycolysis/gluconeogenesis, fructose and mannose metabolism, and pyruvate metabolism. Carbohydrates serve as the primary energy source essential for the growth and development of animals [34]. They are digested in the intestine into monosaccharides, primarily glucose, galactose, and fructose, which are then absorbed into the bloodstream through the small intestine. In this study, we observed that the MGA gene (maltase-glucoamylase) was upregulated in the carbohydrate digestion and absorption pathway of heat-tolerant shrimp, while the SI (sucrase-isomaltase) and G6pt (glucose-6-phosphatase) genes were downregulated, indicating the crucial role of these enzymes in energy supply for coping with high-temperature stress [35].

In addition to carbohydrates, dietary proteins are absorbed as amino acids, serving as an energy reserve. Under high-temperature stress, energy demands rise sharply, and these amino acids can quickly provide energy [36]. In terms of energy supply, glycolysis/gluconeogenesis, fructose and mannose metabolism, and pyruvate metabolism are key pathways for energy metabolism and direct energy acquisition. We found that in the glycolysis/gluconeogenesis pathway of heat-tolerant shrimp, CG7070 (pyruvate kinase-like isoform X2), CG6058 (fructose-bisphosphate aldolase-like isoform X1), and CG10160 (L-lactate dehydrogenase-like isoform X1) were upregulated, while Aldh2 (aldehyde dehydrogenase, mitochondrial-like), Aldh9a1 (4-trimethylaminobutyraldehyde dehydrogenase-like), and CG17725 (phosphoenolpyruvate carboxykinase) were downregulated. The activation of pyruvate kinase and L-lactate dehydrogenase reflects increased glycolytic/glycolytic activity, which facilitates the breakdown of glycogen in the liver into glucose and its transport to osmoregulatory tissues such as the gills and kidneys to provide energy for thermoregulation [37,38]. In fructose and mannose metabolism, QccE-15018 (mannose-6-phosphate isomerase-like), CG32849 (hexokinase type 2 isoform X1), and PFKFB2 (fructose-2,6-bisphosphate/fructose-2,6-bisphosphate kinase-like isoform X1) were upregulated, while Sdh1 (sorbitol dehydrogenase-like), Fuk (putative L-fucose kinase-like), and SORD (sorbitol dehydrogenase-like) were downregulated. In summary, our findings indicate that *P. monodon* adjusts its entire energy supply chain to adapt to high-temperature environments, from the digestion and absorption of energy substrates to the activation of multiple metabolic pathways, ultimately meeting energy demands in elevated temperature conditions.

### 4.2. The Regulation of Innate Immune System Is Necessary for the Black Tiger Shrimp to Adapt to High-Temperature Stress

In this study, the C-type lectin receptor and IL-17 signaling pathway were significantly enriched in high-temperature stress conditions. C-type lectin receptors (CLRs) serve as pattern recognition receptors (PRRs) for pathogen-derived ligands in dendritic cells, macrophages, and neutrophils. Upon ligand binding, CLRs initiate intracellular signaling cascades that induce the production of inflammatory cytokines and chemokines, thereby triggering both innate and adaptive immune responses against pathogens [39,40,41]. The C-lectin of *P. monodon* has been confirmed to combine with bacterial polysaccharides and agglutinate multiple marine pathogenic bacteria, such as *Vibrio harveyi, V. anguillarum, V. alginolyticus*, and so on, to defend against bacterial infection [24]. The high expression of the C-lectin pathway in heat-tolerant shrimp could protect it from bacterial disease. IL-17 is a proinflammatory cytokine produced by activated T-cells and certain cell types of the innate immune system and induces the production of proinflammatory proteins, including cytokines, chemokines, and matrix metalloproteases, to amplify inflammation [42,43]. The IL family is widely present in vertebrates. There are few studies in invertebrate shrimp. The first IL-like gene reported in *L. vannamei* affects antiviral and antibacterial immune responses. The lectin MjCC-CL of kuruma shrimp (*M. japonicus*) contains an IL-like domain, which can recognize bacterial surface molecules and induce antimicrobial peptides to inhibit bacterial infection [44].

The KEGG pathway analysis focusing on two specific pathways suggests a strong association between them. Based on previous studies, in this research, C-lectin may function similarly to IL-17, activating the expression of related factors through an IL-17 pathway analogous to that in mammals. The transcriptomic analysis revealed a significant enrichment of IL-17, with significant upregulation of Relish, MAPK15, Traf6, and JNK, indicating that high-temperature stress in the hepatopancreas induces IL-17A/C-lectin signaling through receptor binding. Concurrently, Hsp90 maintains the integrity of Act1 at the protein level, and Act1, through interaction with TRAF6, activates NF-κB/Relish and MAPK/JNK signaling pathways. These pathways then transcriptionally regulate cytokines, such as Cox2, or antimicrobial peptides, thereby resisting the invasion of exogenous microorganisms. This result suggests that the body increases susceptibility to bacterial infections such as Vibrio under heat stress and then activates the innate immune system to produce antimicrobial peptides, which protect the shrimp and adapt to high-temperature changes. This finding is consistent with previous research results [40,45].

### 4.3. Amino Acid Metabolism Is Necessary for the Black Tiger Shrimp to Adapt to High-Temperature Stress

As previously discussed, under high-temperature stress, protein degradation into amino acids can serve as an energy source, particularly glycogenic amino acids such as histidine, threonine, glutamine, phenylalanine, arginine, serine, tyrosine, methionine, and isoleucine, which can function as direct energy sources or precursors for gluconeogenesis or ketogenesis [46]. In this study, we observed a significant reduction in the levels of various amino acids, including histidine, phenylalanine, valine, and serine, in *P. monodon* under thermal stress. However, the accumulation of ammonia in the body increases due to transamination or deamination processes that promote gluconeogenesis, posing a serious threat to fish health [47]. To counteract ammonia accumulation, the organism must undergo adaptive metabolic adjustments. Notably, we also found a significant increase in γ-glutamyl glutamate and L-glutamate, suggesting the organism’s efforts to mitigate ammonia buildup. Furthermore, Yang Liu et al. have confirmed that the sustained increase in glutamate and glutamine levels under high-temperature stress may be a critical pathway for reducing ammonia accumulation and alleviating its toxicity in *Brachymystax lenok* during thermal stress [48].

### 4.4. Antioxidant Response and Detoxification Is Necessary for the Black Tiger Shrimp to Adapt to High-Temperature Stress

GSH is a soluble thiol antioxidant abundantly present in cells, playing a critical role in maintaining redox homeostasis and protecting cells and tissues from oxidative stress and various forms of stress. It is closely involved in the regulation of redox signaling pathways and detoxification responses [49,50]. The redox balance in organisms is maintained by components such as peroxidases and mGSH, with GSSG being the oxidized form of GSH. In the process catalyzed by glutathione peroxidase (GSH-PX), GSH is oxidized to GSSG while reducing H_2_O_2_ to H_2_O; subsequently, glutathione reductase uses NADPH to regenerate GSH from GSSG [51]. A research report indicates that glutathione metabolism plays a crucial role in the response of aquatic animals to environmental stress. For example, an enhanced hepatic glutathione metabolic response was found in fish under heavy metal exposure [52]. During high-temperature stress, the activities of GST, GSH, and various antioxidant enzymes undergo significant changes, and supplementation with GSH effectively mitigates the impact of high-temperature stress on fish metabolic mechanisms [53,54,55].

This study further reveals significant changes in the expression of glutathione metabolism-related genes in heat-tolerant shrimp, including GSR, TryR, DHAR, GGCT, G6PD, and GGT. The downregulation of genes such as GSR and DHAR limits the interconversion between GSH and GSSG, while the upregulation of GGCT and GGT promotes the conversion of GSH to L-glutamate and L-amino acids. These findings suggest that glutathione in heat-tolerant shrimp operates in a low-sensitivity mode, further supporting the idea that the activity of the glutathione metabolic pathway can serve as an indicator of the heat tolerance level in *P. monodon*. Additionally, the levels of metabolic products such as L-glutamate and L-γ-glutamyl-L-amino acids are also regulated, which may be critical for the thermal adaptation of *P. monodon*.

## 5. Conclusions

**Figure 8:**
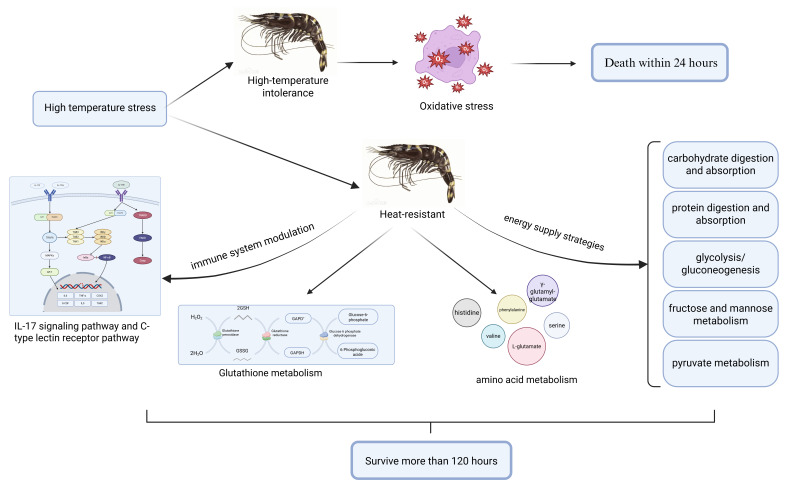
Response pattern of *P. monodon* to high temperature stress.

In this study, through a comprehensive analysis of transcriptomics and metabolomics, we have gained a detailed understanding of the gene expression regulation and metabolic changes associated with thermal tolerance in *P. monodon*. The mechanisms underlying the high-temperature adaptability of this species are complex and involve multiple processes, including energy supply strategies, immune system modulation, amino acid metabolism, and glutathione metabolism (Figure 8). Within the energy supply strategies, pathways related to carbohydrate digestion and absorption, protein digestion and absorption, glycolysis/gluconeogenesis, fructose and mannose metabolism, and pyruvate metabolism collectively regulate energy demands under high-temperature conditions. The immune system’s regulation involves the C-type lectin receptor pathway and the IL-17 signaling pathway, which work together to enhance both innate and adaptive immunity, thereby preventing pathogen invasion. Additionally, various glycogenic amino acids such as histidine, phenylalanine, valine, and serine are metabolized to supply energy, while excess ammonia is converted into γ-glutamyl-glutamate and L-glutamate to mitigate ammonia accumulation. Furthermore, our combined transcriptomic and metabolomic analyses reveals that glutathione metabolism plays a significant role in the adaptation of *P. monodon* to high-temperature thermal stress environments. In summary, this study elucidates the high-temperature tolerance mechanisms in *P. monodon* through investigations of gene expression regulation and metabolic modulation. We have identified key pathways, genes, and metabolites closely associated with thermotolerance traits. While most of these pathways have been reported in thermal stress responses of other aquatic species, the specific expression patterns of genes and metabolites within these pathways exhibit distinct characteristics in *P. monodon.* Collectively, our findings provide both scientific rationale and fundamental data for breeding novel heat-tolerant *P. monodon* varieties. Concurrently, it should be noted that the adaptive mechanisms currently identified necessitate additional experimental verification through well-designed studies. Meanwhile, we are systematically conducting targeted experiments to identify and optimize a set of mechanisms particularly adapted for high-temperature breeding applications.

## Figures and Tables

**Table 1:** List of the genes with a few significant changed KEGG pathways.

Pathway/Gene Symbol	Gene All Name	log2FoldChange	*p*-Value	Up/Down
Glutathione metabolism				
*GLCL*	glutamate-cysteine ligase catalytic subunit-like	1.264	0.00276	UP
*Ggct*	gamma-glutamylcyclotransferase-like	2.413	0.00943	UP
*GST1*	glutathione S-transferase 1-like	−5.980	1.63 × 10^−8^	DOWN
*ICDH*	Isocitrate dehydrogenase (NADP)	−1.652	0.00183	DOWN
*Rrm1*	ribonucleoside-diphosphate reductase large subunit-like	−7.808	3.03 × 10^−16^	DOWN
Lysosome				
*MLA1*	CD63 antigen-like	4.658	1.98 × 10^−11^	UP
*CTSC*	cathepsin C	4.561	3.72 × 10^−5^	UP
*NPC1*	NPC intracellular cholesterol transporter 1-like	−4.530	0.00011	DOWN
*CTSD*	lysosomal aspartic protease-like	−4.764	6.26 × 10^−10^	DOWN
*GLB1*	beta-galactosidase-like	−4.198	1.22 × 10^−13^	DOWN
Glycolysis/Gluconeogenesis				
*CG10160*	L-lactate dehydrogenase-like isoform X1	3.236	9.52 × 10^−6^	UP
*TEgg056i02.1*	aldoketoreductase-like protein	5.122	0.00208	UP
*G6pt*	glucose-6-phosphatase-like	−6.920	1.24 × 10^−15^	DOWN
*ALDH3*	aldehyde dehydrogenase, dimeric NADP-preferring-like	−1.712	0.00442	DOWN
*Aldhx*	aldehyde dehydrogenase X, mitochondrial-like isoform X2	−4.764	3.61 × 10^−10^	DOWN
Glycine, serine, and threonine metabolism				
*Tdh*	L-threonine 3-dehydrogenase, mitochondrial-like	5.340	0.00113	UP
*LPIPOX*	peroxisomal sarcosine oxidase-like	2.781	0.00026	UP
*bhmt*	betaine--homocysteine S-methyltransferase 1-like	−6.325	2.47 × 10^−10^	DOWN
*DAO*	D-amino-acid oxidase-like	−2.561	3.36 × 10^−8^	DOWN
*AGT1*	serine--pyruvate aminotransferase-like isoform X1	−2.175	7.15 × 10^−6^	DOWN
C-type lectin receptor signaling pathway				
*COX2*	cyclooxygenase	5.416	3.55 × 10^−8^	UP
*CG12559*	ERK	4.871	0.01191	UP
*FYN*	tyrosine-protein kinase SRK3-like	5.898	0.00017	UP
*DDB_G0277917*	putative calmodulin-like	−1.112	0.00971	DOWN
*MLT*	putative mucosa-associated lymphoid tissue lymphoma translocation protein 1-like	−1.346	0.01094	DOWN
IL-17 signaling pathway				
*CG11992*	NF-κB transcription factor Relish	1.748	0.00083	UP
*mapk15*	putative mitogen-activated protein kinase 15-like	6.033	0.00114	UP
*Traf6*	tumor necrosis factor receptor-associated factor 6	2.107	0.00236	UP
*CG5680*	c-jun N-terminal kinase (JNK)	2.065	0.00745	UP
*T05E11.3*	Endoplasmin (Hsp90b1)	−1.408	0.00224	DOWN

## Data Availability

The authors will provide the raw data supporting the conclusions of this paper without reservation.

## References

[1] Pörtner H.O., Knust R. (2007). Climate change affects marine fishes through the oxygen limitation of thermal tolerance. Science.

[2] Yu Y., Chen M., Lu Z., Liu Y., Li B., Gao Z., Shen Z. (2022). High-temperature stress will put the thermo-sensitive teleost yellow catfish (*Tachysurus fulvidraco*) in danger through reducing reproductivity. Ecotoxicol. Environ. Saf..

[3] Huang T., Gu W., Liu E., Wang B., Wang G., Dong F., Guo F., Jiao W., Sun Y., Wang X. (2022). miR-301b-5p and its target gene nfatc2ip regulate inflammatory responses in the liver of rainbow trout (*Oncorhynchus mykiss*) under high temperature stress. Ecotoxicol. Environ. Saf..

[4] Li H., Yu H., Zhang X., Huang W., Zhang C., Wang C., Gao Q., Dong S. (2024). Temperature acclimation improves high temperature tolerance of rainbow trout (*Oncorhynchus mykiss*) by improving mitochondrial quality and inhibiting apoptosis in liver. Sci. Total Environ..

[5] Lim M.Y., Bernier N.J. (2023). Intergenerational plasticity to cycling high temperature and hypoxia affects offspring stress responsiveness and tolerance in zebrafish. J. Exp. Biol..

[6] Lee D., Kim K., Park J., Lee J., Kim J. (2023). High water temperature-mediated immune gene expression of olive flounder, *Paralichthys olivaceus* according to pre-stimulation at high temperatures. Environ. Toxicol. Pharmacol..

[7] Huang Z., Guo X., Wang Q., Ma A., Zhao T., Qiao X., Li M. (2022). Digital RNA-seq analysis of the cardiac transcriptome response to thermal stress in turbot Scophthalmus maximus. J. Therm. Biol..

[8] Xiong D., Duan Y., Xu J., Zhan A., Chen C., Zhang J. (2020). Physiological responses in gills of *Litopenaeus vannamei* exposed to the combined stress of temperature and ammonia. J. South. Agric..

[9] Zhu M., Yao C. (2015). The impact of temperature on the oxygen metabolism and energy metabolism in the hepatopancreas of shrimp *Litopenaeus vannamei*. J. Fish. China.

[10] Zhu X., Zheng X., Xing Y., Huang J., Dong H., Zhang J. (2024). Study on tributyrin enhancing anti periodic high temperature stress ability of gill tissue in *Litopenaeus vannamei*. South China Fish. Sci..

[11] Earhart M.L., Blanchard T.S., Harman A.A., Schulte P.M. (2022). Hypoxia and High Temperature as Interacting Stressors: Will Plasticity Promote Resilience of Fishes in a Changing World?. Biol. Bull..

[12] Heise K., Puntarulo S., Nikinmaa M., Abele D., Pörtner H. (2006). Oxidative stress during stressful heat exposure and recovery in the North Sea eelpout *Zoarces viviparus* L.. J. Exp. Biol..

[13] González-Ruiz R., Leyva-Carrillo L., Peregrino-Uriarte A.B., Yepiz-Plascencia G. (2023). The combination of hypoxia and high temperature affects heat shock, anaerobic metabolism, and pentose phosphate pathway key components responses in the white shrimp (*Litopenaeus vannamei*). Cell Stress Chaperon.

[14] Ndong D., Chen Y., Lin Y., Vaseeharan B., Chen J. (2007). The immune response of tilapia *Oreochromis mossambicus* and its susceptibility to *Streptococcus iniae* under stress in low and high temperatures. Fish Shellfish Immun..

[15] Huang D., Ren M., Liang H., Ge X., Xu H., Wu L. (2022). Transcriptome analysis of the effect of high-temperature on nutrient metabolism in juvenile grass carp (*Ctenopharyngodon idellus*). Gene.

[16] Zhang X., Yuan J., Zhang X., Yu Y., Li F. (2022). Comparative transcriptomic analysis unveils a network of energy reallocation in *Litopenaeus vannamei* responsive to heat-stress. Ecotoxicol. Environ. Saf..

[17] Luo L., Huang J., Liu D., Jiang S., Zhou F., Jiang S., Yang Q., Li Y., Li T., Tan L. (2022). Comparative transcriptome analysis of differentially expressed genes and pathways in *Procambarus clarkii* (*Louisiana crawfish*) at different acute temperature stress. Genomics.

[18] Viant M.R. (2008). Recent developments in environmental metabolomics. Mol. Biosyst..

[19] Fang M., Lei Z., Ruilin M., Jing W., Leqiang D. (2023). High temperature stress induced oxidative stress, gut inflammation and disordered metabolome and microbiome in tsinling lenok trout. Ecotoxicol. Environ. Saf..

[20] Li L., Liu Z., Quan J., Lu J., Zhao G., Sun J. (2022). Metabonomics analysis reveals the protective effect of nano-selenium against heat stress of rainbow trout (*Oncorhynchus mykiss*). J. Proteom..

[21] Chen Y., Wu X., Lai J., Liu Y., Song M., Li F., Gong Q. (2023). Integrated biochemical, transcriptomic and metabolomic analyses provide insight into heat stress response in Yangtze sturgeon (*Acipenser dabryanus*). Ecotoxicol. Environ. Saf..

[22] Zhao T., Ma A., Yang S., Huang Z. (2021). Integrated metabolome and transcriptome analyses revealing the effects of thermal stress on lipid metabolism in juvenile turbot *Scophthalmus maximus*. J. Therm. Biol..

[23] Huang J.H. (2018). Penaeus Monodon Breeding and Culture Technology.

[24] Qin Y., Jiang S., Huang J., Zhou F., Yang Q., Jiang S., Yang L. (2019). C-type lectin response to bacterial infection and ammonia nitrogen stress in tiger shrimp (*Penaeus monodon*). Fish Shellfish Immun..

[25] Jiravanichpaisal P., Söderhäll K., Söderhäll I. (2004). Effect of water temperature on the immune response and infectivity pattern of white spot syndrome virus (WSSV) in freshwater crayfish. Fish Shellfish Immun..

[26] Magouz F.I., Moustafa E.M., Abo-Remela E.M., Halawa M.R., Barakaat P.M., Omar A.A. (2024). Summer mortality syndrome bacterial pathogens in farmed Nile tilapia (*Oreochromis niloticus*). Open Vet. J..

[27] Zhang X., Sun J., Chen F., Qi H., Chen L., Sung Y.Y., Huang Y., Lv A., Hu X. (2021). Phenotypic and genomic characterization of a Vibrio parahaemolyticus strain causing disease in *Penaeus vannamei* provides insights into its niche adaptation and pathogenic mechanism. Microb. Genom..

[28] Nandy T., Baag S., Mandal S. (2021). Impact of elevated temperature on physiological energetics of *Penaeus monodon* post larvae: A mesocosm study. J. Therm. Biol..

[29] Fan R., Li Y., Jiang S., Jiang S., Yang Q., Yang L., Huang J., Zhou F. (2021). cDNA cloning and expression analysis of glutaredoxin 3 in black tiger shrimp *Penaeus monodon*. Aquac. Int..

[30] Liu B., Xie J., Ge X., Xu P., Wang A., He Y., Zhou Q., Pan L., Chen R. (2010). Effects of anthraquinone extract from Rheum officinale Bail on the growth performance and physiological responses of *Macrobrachium rosenbergii* under high temperature stress. Fish Shellfish Immunol..

[31] Madeira C., Leal M.C., Diniz M.S., Cabral H.N., Vinagre C. (2018). Thermal stress and energy metabolism in two circumtropical decapod crustaceans: Responses to acute temperature events. Mar. Environ. Res..

[32] Wang X., Wang L., Zhang H., Ji Q., Song L., Qiu L., Zhou Z., Wang M., Wang L. (2012). Immune response and energy metabolism of *Chlamys farreri* under *Vibrio anguillarum* challenge and high temperature exposure. Fish Shellfish Immun..

[33] Zhang G., Li L., Meng J., Qi H., Qu T., Xu F., Zhang L. (2016). Molecular Basis for Adaptation of Oysters to Stressful Marine Intertidal Environments. Annu. Rev. Anim. Biosci..

[34] Liu H., Pan L., Shen J., Tan B., Dong X., Yang Q., Chi S., Zhang S. (2023). Effects of Carbohydrase Supplementation on Growth Performance, Intestinal Digestive Enzymes and Flora, Glucose Metabolism Enzymes, and glut2 Gene Expression of Hybrid Grouper (*Epinephelus fuscoguttatus*♀ × *E. lanceolatus*♂) Fed Different CHO/L Ratio Diets. Metabolites.

[35] Blanco A.M., Bertucci J.I., Sánchez-Bretaño A., Delgado M.J., Valenciano A.I., Unniappan S. (2017). Ghrelin modulates gene and protein expression of digestive enzymes in the intestine and hepatopancreas of goldfish (*Carassius auratus*) via the GHS-R1a: Possible roles of PLC/PKC and AC/PKA intracellular signaling pathways. Mol. Cell. Endocrinol..

[36] Andersen S.M., Waagbø R., Espe M. (2016). Functional amino acids in fish nutrition, health and welfare. Front. Biosci..

[37] Jiang J., Xu J., Ye L., Sun M., Jiang Z., Mao M. (2020). Identification of differentially expressed genes in gills of tiger puffer (*Takifugu rubripes*) in response to low-salinity stress. Comp. Biochem. Physiol. Part B Biochem. Mol. Biol..

[38] Liu J., Jin P., Li M., Yi X., Tian Y., Zhang Z., Liu J., Shi L. (2024). The energy metabolism of the freshwater leech *Whitmania pigra* in response to fasting. Comp. Biochem. Physiol. Part B Biochem. Mol. Biol..

[39] Li Y., Pan L., Yu J. (2022). The injection of one recombinant C-type lectin (LvLec) induced the immune response of hemocytes in *Litopenaeus vannamei*. Fish Shellfish Immun..

[40] Sun J., Lan J., Zhao X., Vasta G.R., Wang J. (2017). Binding of a C-type lectin’s coiled-coil domain to the Domeless receptor directly activates the JAK/STAT pathway in the shrimp immune response to bacterial infection. PLoS Pathog..

[41] Wang X., Vasta G.R., Wang J. (2020). The functional relevance of shrimp C-type lectins in host-pathogen interactions. Dev. Comp. Immunol..

[42] Mcgeachy M.J., Cua D.J., Gaffen S.L. (2019). The IL-17 Family of Cytokines in Health and Disease. Immunity.

[43] Amatya N., Garg A.V., Gaffen S.L. (2017). IL-17 Signaling: The Yin and the Yang. Trends Immunol..

[44] Liang Q., Zheng J., Zuo H., Li C., Niu S., Yang L., Yan M., Weng S., He J., Xu X. (2017). Identification and characterization of an interleukin-16-like gene from pacific white shrimp *Litopenaeus vannamei*. Dev. Comp. Immunol..

[45] Chang S.H., Dong C. (2011). Signaling of interleukin-17 family cytokines in immunity and inflammation. Cell. Signal..

[46] Kumar V., Sahu N.P., Pal A.K., Kumar S., Sinha A.K., Ranjan J., Baruah K. (2010). Modulation of key enzymes of glycolysis, gluconeogenesis, amino acid catabolism, and TCA cycle of the tropical freshwater fish *Labeo rohita* fed gelatinized and non-gelatinized starch diet. Fish Physiol. Biochem..

[47] Xu Z., Cao J., Qin X., Qiu W., Mei J., Xie J. (2021). Toxic Effects on Bioaccumulation, Hematological Parameters, Oxidative Stress, Immune Responses and Tissue Structure in Fish Exposed to Ammonia Nitrogen: A Review. Animals.

[48] Liu Y., Liu J., Ye S., Bureau D.P., Liu H., Yin J., Mou Z., Lin H., Hao F. (2019). Global metabolic responses of the lenok (*Brachymystax lenok*) to thermal stress. Comp. Biochem. Physiol. Part D Genom. Proteom..

[49] Forman H.J., Zhang H., Rinna A. (2009). Glutathione: Overview of its protective roles, measurement, and biosynthesis. Mol. Asp. Med..

[50] Wang L., Ahn Y.J., Asmis R. (2020). Sexual dimorphism in glutathione metabolism and glutathione-dependent responses. Redox Biol..

[51] Calabrese G., Morgan B., Riemer J. (2017). Mitochondrial Glutathione: Regulation and Functions. Antioxid. Redox Sign..

[52] Eroglu A., Dogan Z., Kanak E.G., Atli G., Canli M. (2015). Effects of heavy metals (Cd, Cu, Cr, Pb, Zn) on fish glutathione metabolism. Environ. Sci. Pollut. Res. Int..

[53] Kim S., Kim A., Ma S., Lee W., Lee S., Yoon D., Kim D., Kim S. (2019). Glutathione Injection Alleviates the Fluctuation of Metabolic Response under Thermal Stress in Olive Flounder, *Paralichthys olivaceus*. Metabolites.

[54] Mahanty A., Purohit G.K., Banerjee S., Karunakaran D., Mohanty S., Mohanty B.P. (2016). Proteomic changes in the liver of Channa striatus in response to high temperature stress. Electrophoresis.

[55] Schleger I.C., Pereira D.M.C., Resende A.C., Romão S., Herrerias T., Neundorf A.K.A., de Souza M.R.D.P., Donatti L. (2024). Metabolic responses in the gills of Yellowtail Lambari Astyanax lacustris under low- and high-temperature thermal stress. J. Aquat. Anim. Health.

